# *De Novo* Polymerase Activity and Oligomerization of Hepatitis C Virus RNA-Dependent RNA-Polymerases from Genotypes 1 to 5

**DOI:** 10.1371/journal.pone.0018515

**Published:** 2011-04-07

**Authors:** Pilar Clemente-Casares, Alberto J. López-Jiménez, Itxaso Bellón-Echeverría, José Antonio Encinar, Elisa Martínez-Alfaro, Ricardo Pérez-Flores, Antonio Mas

**Affiliations:** 1 Centro Regional de Investigaciones Biomédicas (CRIB), Universidad de Castilla La Mancha, Albacete, Spain; 2 Infectious Disease Unit, Complejo Hospitalario Universitario de Albacete, Albacete, Spain; 3 Digestive Department, Complejo Hospitalario Universitario de Albacete, Albacete, Spain; 4 Instituto de Biología Molecular y Celular, Universidad Miguel Hernández, Elche, Spain; Louisiana State University Health Sciences Center, United States of America

## Abstract

Hepatitis C virus (HCV) shows a great geographical diversity reflected in the high number of circulating genotypes and subtypes. The response to HCV treatment is genotype specific, with the predominant genotype 1 showing the lowest rate of sustained virological response. Virally encoded enzymes are candidate targets for intervention. In particular, promising antiviral molecules are being developed to target the viral NS3/4A protease and NS5B polymerase. Most of the studies with the NS5B polymerase have been done with genotypes 1b and 2a, whilst information about other genotypes is scarce. Here, we have characterized the *de novo* activity of NS5B from genotypes 1 to 5, with emphasis on conditions for optimum activity and kinetic constants. Polymerase cooperativity was determined by calculating the Hill coefficient and oligomerization through a new FRET-based method. The V_max_/K_m_ ratios were statistically different between genotype 1 and the other genotypes (p<0.001), mainly due to differences in V_max_ values, but differences in the Hill coefficient and NS5B oligomerization were noted. Analysis of sequence changes among the studied polymerases and crystal structures show the αF helix as a structural component probably involved in NS5B-NS5B interactions. The viability of the interaction of αF and αT helixes was confirmed by docking studies and calculation of electrostatic surface potentials for genotype 1 and point mutants corresponding to mutations from different genotypes. Results presented in this study reveal the existence of genotypic differences in NS5B *de novo* activity and oligomerization. Furthermore, these results allow us to define two regions, one consisting of residues Glu128, Asp129, and Glu248, and the other consisting of residues of αT helix possibly involved in NS5B-NS5B interactions.

## Introduction

The hepatitis C virus (HCV) is a positive-strand RNA ((+)RNA) virus with a high-titer and error-prone replication rate leading to the generation of viral populations in which mixtures of almost infinite different variants may coexist [1], [2], [3]. HCV infection is widespread worldwide, showing geographical differences in terms of genetic identity with six well defined genotypes [4]–[6]. Important biological and antigenic differences exist between variants, and different genotypes respond differently to treatment [7]. The goal of HCV treatment is to achieve a sustained virological response (SVR) defined as the absence of HCV viral load for at least six months after the end of the treatment. About 80% of patients infected with genotypes 2, 3 and 5 achieve SVR after treatment with pegylated-interferon (PEG-IFN) plus ribavirin, whereas the rate of SVR is roughly 50% among those with genotype 1 and it is somewhat intermediate for genotypes 4 and 6 [8].

HCV replicates its positive sense genome through an RNA intermediate of negative sense [9]. The NS5B protein is responsible for the synthesis of the (+) strand progeny via a (−) strand intermediate through an RNA-dependent RNA-polymerase (RdRp) activity [10],[11]. *In vitro* RNA synthesis by NS5B may be induced in the presence of a template-primer or initiated *de novo*. However, RNA synthesis *in vivo* occurs by a *de novo* mechanism [12],[13]. Manganese has been proposed as the preferred cation for HCV NS5B for the de novo initiation step [12],[14]. Most biochemical and biophysical studies of NS5B have been done with genotype 1b and have disregarded the NS5B polymerase activity of viruses from different genotypes. The biochemical properties of NS5B of other genotypes have been described in the context of NS5B inhibitor studies [15]–[19] without a comprehensive analysis of the polymerase activities of all 6 genotypes [20].

Similarly to other (+)RNA viruses, HCV replicates its genome in, so called, replication complexes (RC), where viral and cellular proteins co-localize. In HCV RC, a large excess of each HCV non-structural protein respect to (+) and (−) strand HCV RNA has been observed [21], suggesting extensive protein-protein interactions and molecular crowding phenomena. HCV NS5B interacts with itself, affecting RNA synthesis activity in a cooperative way [22]. This interaction has been proposed as the target for some of the non-nucleoside inhibitors directed against the NS5B protein [23]–[26].

Here, we have cloned, over-expressed, purified and evaluated NS5B polymerases from different HCV genotypes. Reaction conditions for RdRp activity in a *de novo* initiation assay, and cooperativity have been analyzed for proteins derived for genotypes 1 to 5. Also, we have analyzed NS5B-NS5B interactions *in vitro* by a fluorescence resonance energy transfer (FRET)-based method using HCV polymerases fused with GFP derivatives. Finally, we have performed docking simulations. Our results together with primary amino acid sequences and structural data deposited at the Protein Data Bank have allowed us to define residues probably involved in NS5B-NS5B interactions.

## Materials and Methods

### NS5BΔ21 polymerases, fusion proteins and plasmids construction

Plasmids containing the gene coding regions of HCV NS5B of genotypes 1 to 5 were constructed by molecular cloning. NS5B polymerases were amplified as deletion mutants of the 21 amino acids of their C-terminal end (NS5BΔ21) to avoid the presence of the highly hydrophobic domain and ensure the solubility of the enzyme. NS5B from genotypes 1b and 2a were obtained from plasmids pCVJ4L6S and pJ6CF respectively, kindly provided by Dr. Jens Bukh (University of Copenhagen). NS5B-coding regions from HCV genotypes 3, 4, and 5 were obtained as follows. Briefly, plasma samples from patients infected with HCV of these genotypes were collected and viral RNA was extracted by using the QIAamp Viral RNA Mini Kit (Qiagen, Barcelona, Spain) following the manufacturer instructions. cDNA was synthesized by amplification with genotype-specific antisense primers (Table S1) and a mixture of Superscript™ III Reverse Transcriptase (Invitrogen, El Prat de Llobregat, Spain) and AMV Reverse Transcriptase (Promega, Madrid, Spain) as described by Fan et al [27]. Primers designed to amplify the complete HCV polyprotein coding region were used in a first round of PCR amplification. Then, a nested PCR with internal primers designed to amplify the complete NS5B-coding region was performed and the purified products were cloned into a high copy vector (pSC-B-amp/kan from the Strataclone Blunt PCR Cloning kit, Stratagene, Madrid, Spain), following the manufacturer instructions. DNA of the resulting Δ21 mutants from genotypes 1 to 5 flanked by sequences required for recombination with the Gateway technology (Invitrogen) and a 6xHis tag at the c-terminal were amplified by PCR and introduced into the pDEST14 vector (Invitrogen) for expression in *E. coli* of the corresponding protein following the manufacturer instructions.

NS5BΔ21 from genotypes 1 to 5 were fused to fluorescent proteins (FP), either cyan or citrine, as previously described [23]. All PCR reactions were performed using a high-fidelity polymerase (rTth DNA Polymerase, XL from Applied Biosystems, Madrid, Spain, Expand High Fidelity System from Roche,Valencia, Spain, or Pfu Turbo DNA polymerase from Stratagene). PCR products containing NS5BΔ21-FP fusions were cloned into the pDest14 vector by using the Gateway technology to obtain the final pDest14-NS5BΔ21-FP constructs (NS5BΔ21-cyan and NS5BΔ21-citrine). All constructs were verified by DNA sequencing (Macrogen, Korea).

### Genotyping and phylogenetic analyses

Genotyping was confirmed by using the sequences obtained above. Briefly, multiple sequence alignments were carried out using the CLUSTALX software [28]. Phylogenetic analyses were performed using the neighbor-joining method included in the PHYLIP software package [29]. The robustness of the grouping was determined by bootstrap re-sampling of multiple sequence alignments (100 sets) with the programs SEQBOOT, DNADIST, NEIGHBOR, and CONSENSE. The output graphics of the trees were created with the TREEVIEW software package, version 1.5. Genotype reference sequences were downloaded from *Los Alamos HCV database* [4]. Patient-derived NS5B sequences from genotypes 3, 4 and 5 have been deposited under the GenBank Accession Numbers HM107694, HM107695 and HM107696 respectively.

### Protein over-expression and purification

Proteins of all five genotypes used in this study were over-expressed and purified as previously reported [23]. Aliquots showing purest and most concentrated protein were adjusted to 50% glicerol and stored at −80°C. All purification processes were followed by SDS-PAGE and Coomassie blue staining and quantified by Bradford protein assay and SDS-PAGE gel imaging. Western blots were performed by standard procedures using α-NS5B (Abcam, Cambridge, UK) as the primary antibody.

### Polymerase activity assay and determination of kinetic constants

*De novo* RdRp activity of purified NS5BΔ21s was examined by incorporation of radiolabeled GMP on homopolymeric (polyC) template (average length of 300 residues). The reaction was performed, except when indicated, in polymerase buffer (20 mM MOPS pH 7.25, 66 mM NaCl, 5 mM MnCl_2_, and 40 ng/µl of polyC), in the presence of 125 µM GTP, and 0.5 µCi of α[^32^P]GTP (3000 Ci/mmol, PerkinElmer). Reactions were initiated by the addition of 600 nM purified NS5B and incubated at 25°C. The nominal input concentration of NS5BΔ21 was 600 nM, however, it has been established that only a small fraction (<1%) of NS5BΔ21 enzyme purified from bacteria is catalytically competent *in vitro* [30]. Aliquots were withdrawn over time and reactions were stopped by adding 150 mM EDTA. Reaction products were transferred onto DE81 paper membrane (Whatman International Ltd, Barcelona, Spain). DE81 filter papers were then washed twice with 9 ml of 0.2 mM Na_2_HPO_4_, once with 9 ml of H_2_O and once with 3 ml of absolute ethanol, and dried for 15 min at 55°C. The radioactivity bound to the filter was determined using liquid scintillation counting (LS6500, Beckman Coulter). Polymerase activity of NS5BΔ21-fluorescence proteins was determined using LE19 RNA as template, as previously described [14].

The homopolymeric (polyC) template (average length of 300 residues) was used to analyze the kinetic constants V_max_ and K_m_ for GTP. Briefly, 0.5 µCi of α[^32^P]GTP (3000Ci/mmol, PerkinElmer) and 600 nM of purified NS5B were added to polymerase buffer, and the reactions were started by addition of GTP (6.25, 12.5, 25, 50, 100, 200, 300, 500, and 1000 µM), and incubated at 25°C for 15 minutes. GMP incorporation was measured by liquid scintillation counting as described above. Similar procedures were done to calculate the kinetic parameters for the template (polyC). No attempts were made to titrate the active site concentration of NS5B preparations. Therefore, maximum velocity values (V_max_) related to total enzyme concentration are reported instead of kcat values. To ensure that protein preparations were properly folded and they were active for RNA binding, electro-mobility shift assay for each genotype at two different protein concentrations was also performed as previously described [23].The dependence of Vi on NTP or polyC concentrations are described by a hyperbolic equation from which we can calculate V_max_ and K_m_, the maximal velocity and the affinity constant (for NTP or polyC) by NS5B, respectively. V_max_ and K_m_ were determined from curve-fitting using GraphPad Prism (GraphPad Software Inc).

For calculation of the Hill coefficient, polymerase reactions were performed in polymerase buffer in the presence of 125 µM GTP, and 0.5 µCi of α[^32^P]GTP (3000Ci/mmol, PerkinElmer). The polymerase concentration was increased from 37.5 nM to 1.5 µM. Specific activity was fitted to a sigmoidal curve using the equation log(v/[V_max_ - v]) = h log[E] - log K, described by Copeland [31], where v is the velocity at a given enzyme concentration [E] and h is the Hill coefficient. h values above 1 indicate positive cooperative RNA synthesis.

### Fluorescence spectroscopic and FRET analyses

Fluorescence spectroscopic analyses for each NS5BΔ21-FP individually (50 nM final concentration) as well as FRET analyses for mixtures of NS5BΔ21-cyan and NS5BΔ21-citrine of each genotype (50 nM final concentration of each protein) were performed as previously described [23]. For fluorescence spectroscopic analyses of NS5BΔ21-cyan, the excitation wavelength (λ_ex_) was set at 432 nm (excitation wavelength for cyan) and the fluorescence emission spectra were obtained from 460 to 600 nm. For NS5BΔ21-citrine, λ_ex_ was set at 460 nm (excitation wavelength for citrine) and the fluorescence emission spectra were obtained from 500 to 600 nm. For FRET analyses (mixtures of NS5BΔ21-cyan and NS5BΔ21-citrine), λ_ex_ was set at 432 nm to obtain fluorescence emission spectra from 460 to 600 nm. Spectra were recorded at 1200 nm/min using a five nm slit and the photomultiplier set at 600 V. If FRET conditions are optimal, then fluorescence increases at 530 nm (F530 citrine emission, FRET signal), while fluorescence at 478 nm decreases (F478 primary cyan emission) with an isosbestic wavelength at ∼512 nm. As negative control, we subtracted the spectra obtained in the presence of NS5BΔ21-citrine alone because citrine cross-excites slightly at 432 nm. Finally, the data were used to calculate a simple ratio of FRET (emission at 530 nm/emission at 478 nm). Fluorescence measurements were obtained in a Hitachi F-7000 Fluorescence Spectrophotometer.

### Electrostatic surface potential

Adaptative Poisson-Boltzmann Solver is a software that calculates numerical solutions of the Poisson-Boltzmann equation, describing electrostatic interactions between molecular solutes in salty, aqueous media. The color coded electrostatic surface potential for NS5B was drawn using the Adaptive Poisson-Boltzmann Solver package [32] within PYMOL 1.3 [33] using 2ZKU.pdb as template for building a protein-protein complex. In silico point mutations in the F helix for the different genotypes were performed with FoldX [34] and 2ZKU.pdb as template. Energy minimization for all the structures that were involved in these studies was done using the FoldX software.

### Docking studies

The GRAMM-X Protein Docking Web Server v.1.2.0 [35] was used for docking the NS5B with itself. The output PDB file contains the desired number of models (between 1 and 300) ranked as the most probable prediction candidates according to the scoring function used by the program. The same 2ZKU.pdb acted as receptor and ligand in this study of oligomerization. We used positions His502 to Arg508 as receptor residues that might form the interface with the ligand, and position Asp125 to Asp129 as potential ligand interface residues. To select a model out of the top scoring docked complexes reported by GRAMM-X, we calculated the binding energy on the generated complexes removing intraclashes (using the strongest van der Waals parameters) with FoldX [34]. Ionic protein-protein interactions were studied with the Protein Interaction Calculator (PIC) web server [36].

## Results

### PCR amplification of NS5B-coding sequences and genotyping

**Figure 1:**
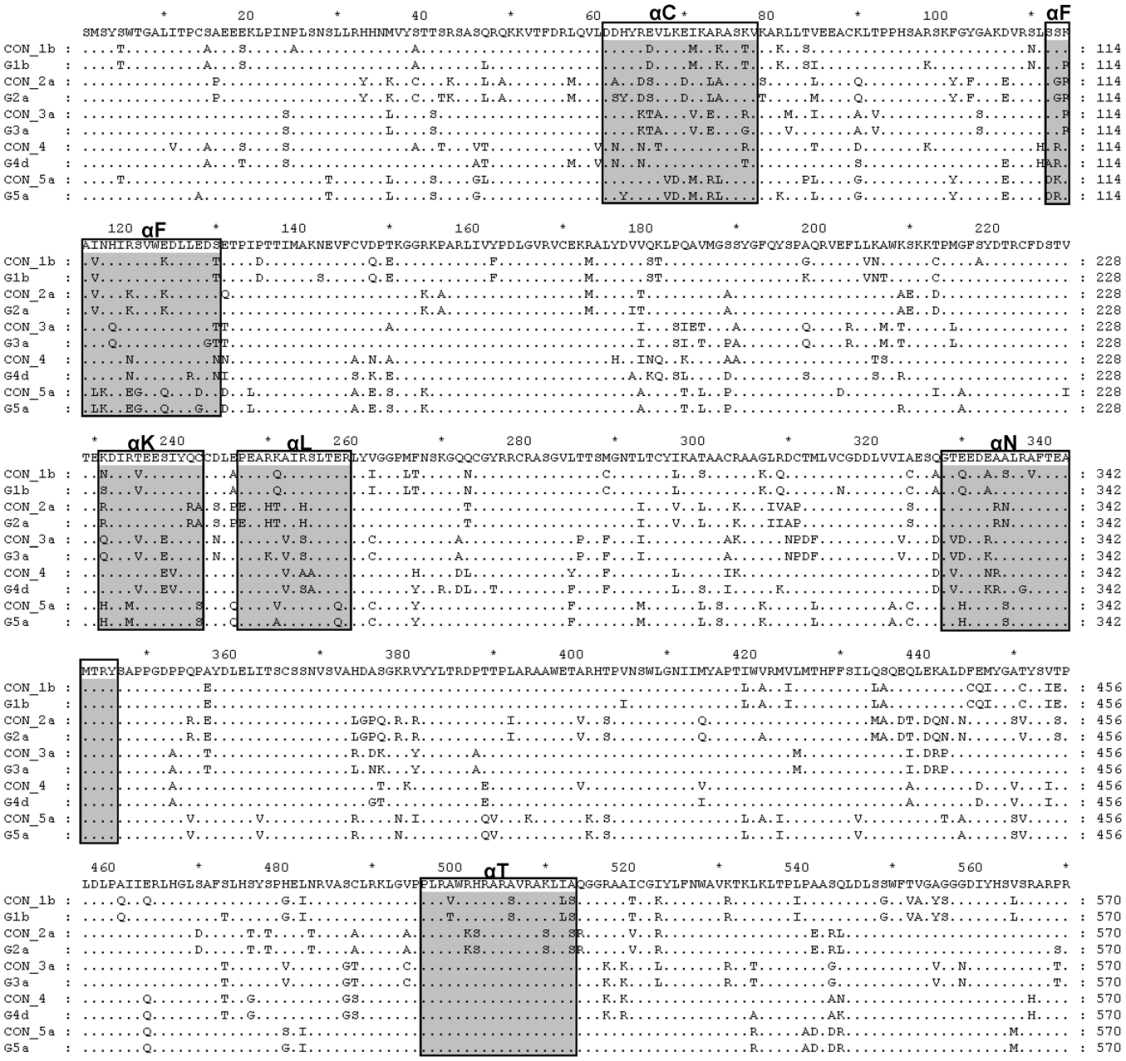
Amino acid sequences of the isolates used in this study. Alignment of the predicted amino acid sequences (Δ21) of the NS5B proteins used in this study and the consensus sequences corresponding to subtypes 1b, 2a, 3a and 5a. As only 1 sequence for subtype 4d was present in the http://hcv.lanl.gov/the consensus for NS5BΔ-4d corresponds to the whole genotype 4. Only differences with a general consensus sequence are shown. Gray boxes indicate the sequences corresponding to αhelixes C, F, K, L, and N.

**Figure 2:**
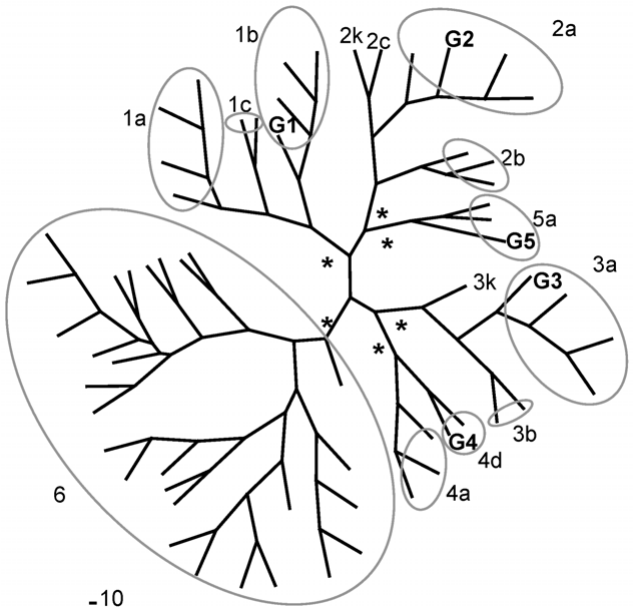
Phylogenetic analysis. Neighbor-joining phylogram of NS5BΔ21 from this study (G1, G2, G3, G4 and G5) and the same reference sequences as in A). A consensus for genotype 6 has also been included. Bootstrap analysis was performed with 100 repetitions. *  =  bootstrap values of 100% showing the divergence of the different genotypes.

Viral RNAs from plasma of patients infected with HCV genotypes 3, 4 and 5 were used as templates for RT-PCRs. Genotype-derived amino acid sequences corresponding to the NS5BΔ21 used in this study (named G1b, G2a, G3a, G4d and G5a) were aligned together with consensus sequences from each genotype (named CON_#genotype) (Fig. 1). Amino acid identity among genotypes was around 75–82%. Nucleotide (Kimura 2-parameters) and protein (Dayhoff PAM method) distances among the different NS5B sequences and the consensus for each genotype are shown in Table 1. We found distances up to 45% and 36% for nucleotide and amino acid sequences, respectively. Phylogenetic analyses using reference sequences confirmed the genotype and subtype of the isolates used in this study (Fig. 2).

**Table 1:** Genetic distances.

	G1bΔ21	G2aΔ21	G3aΔ21	G4dΔ21	G5aΔ21	CON_1b	CON_2a	CON_3a	CON_4	CON_5a
G1bΔ21	-	33.50	31.49	29.60	31.81	4.20	33.19	31.07	28.43	31.68
G2aΔ21	42.21	-	34.10	36.25	31.07	34.00	2.09	34.15	34.02	31.88
G3aΔ21	41.37	44.14	-	23.07	30.81	32.26	33.25	1.95	23.91	32.77
G4dΔ21	34.71	42.40	34.75	-	29.02	29.06	34.17	23.58	10.27	31.64
G5aΔ21	38.80	41.31	39.88	40.30	-	29.44	29.53	30.95	30.74	3.33
CON_1b	5.13	42.81	41.04	34.90	37.71	-	33.63	32.35	29.56	30.19
CON_2a	42.83	4.76	43.77	41.73	42.23	42.91	-	33.37	32.73	30.10
CON_3a	39.39	45.25	3.95	35.40	41.28	39.55	44.88	-	23.50	32.89
CON_4	36.92	41.81	34.49	15.64	38.32	37.22	41.20	34.81	-	32.74
CON_5a	39.61	41.08	41.65	40.26	7.54	38.26	41.89	42.87	39.61	-

Nucleotide (below diagonal) and protein (above diagonal) distance matrix in percentage obtained by the Kimura 2-parameters and the Dayhoff PAM method, respectively, between the NS5B sequences used in this study and consensus sequences obtained for the 5 major genotypes.

### Purification of NS5BΔ21 proteins from different genotypes

**Figure 3:**
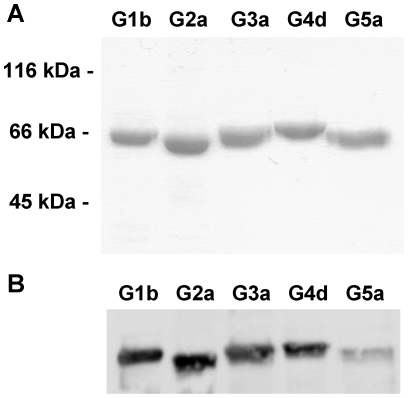
Purification of NS5BΔ21 proteins by affinity chormatography and cationic exchange. A) Purified proteins after cationic exchange chromatography (heparin-sepharose column) eluted at 500 mM NaCl and detected with Coomassie blue staining. B) Western blot detection of HCV NS5B genotypes 1 to 5 with a polyclonal antibody. Protein molecular weigth markers (in kilodaltons) are shown on the left.

HCV NS5BΔ21 proteins containing a 6x histidine tag at the C-terminal were over-expressed and purified to apparent homogeneity, as judged by SDS-PAGE and Coomassie blue staining. The electrophoretic mobility of the purified NS5BΔ21 proteins was compatible with their deduced molecular mass (63.2–63.4 kDa), although with small differences among genotypes (Fig. 3A). Typical protein yields ranged from 2 to 10 mg. Bands corresponding to NS5BΔ21for all five genotypes were further identified by Western blot analyses with a polyclonal antibody directed against the NS5B from genotype 1b (Fig. 3B). Genotype 5 showed the weakest band, probably due to sequence changes in the antibody recognition sites. Fusion proteins (NS5BΔ21-FPs) for FRET studies were also purified and the electrophoretic mobility was also compatible with their deduced molecular mass (90 KDa approximately, data not shown).

### Biochemical properties of NS5BΔ21 from different genotypes

The V_max_ and K_m_ constants for GTP were calculated for HCV NS5BΔ21 polymerases from all 5 genotypes and the results are shown in Table 2. The affinity of NS5BΔ21for GTP showed small differences among genotypes and ranged from almost 100 µM to 208 µM. However, V_max_ values showed great differences among them. Genotype 1 NS5BΔ21 showed the highest V_max_ which was 4-fold higher than that of genotype 3, almost 7-fold higher than those of genotypes 2 and 5, and more than 20–fold higher than the calculated V_max_ for NS5BΔ21 from genotype 4. Therefore, the V_max_/K_m_ ratio decreased approximately 6-, 7.5- and 8- fold for genotypes 2, 3 and 5 respectively, and 34-fold for genotype 4 with respect to genotype 1. In contrast, the template kinetic constants (polyC in our studies) did not show significant differences among polymerases from different genotypes, with K_m_ values ranging from 53 nM to 119 nM (Table 2).

**Table 2:** Kinetic constants and Hill coefficients of HCV NS5B polymerase of different genotypes.

Genotype	Kinetic constants				Hill coefficient
	GTP			Template (polyC)^a^	
	K_m_ (µM)	V_max_ (pmoles/min)	V_max_/K_m_	K_m_ (nM)	
NS5BΔ21-1b	117.6±24.40	14.3±1.242	0.122	95.54±18.57	2.9±0.6
NS5BΔ21-2a	99.44±22.96	2.53±0.187	0.020	57.52±16.96	1.9±0.3
NS5BΔ21-3a	208.6±40.3	3.23±0.316	0.015	118.9±22.80	3.8±0.6
NS5BΔ21-4d	184.54±4.69	0.66±0.0594	3.59x10^−3^	53.24±11.39	2.6±0.2
NS5BΔ21-5a	134.6±41,67	2.18±0.215	0.016	65.47±25.11	-

a Referred to 5′ ends.

Electro-mobility shift assay experiments for each genotype at two different protein concentrations were performed as described in Materials and Methods. We did not find substantial differences between genotypes (Figure S1A). At the NS5B concentration at which the RNA polymerase activity was determined (600 nM) the ratio of protein-bound to free RNA probe was 1.12, 0.59, 0.72, 1.05 and 0.63 for genotypes 1, 2, 3, 4, and 5, respectively (Figure S1B). The value V_max_/K_m_ was calculated normalized with regard to this ratio and is also shown (Figure S1C). Results with and without normalization were very similar and three groups could be defined, being genotype 1b the most efficient, genotypes 2, 3, and 5 showing an intermediate efficiency, and genotype 4 the less efficient RNA-polymerase.

### Effect of the reaction conditions on polymerase activity

**Figure 4:**
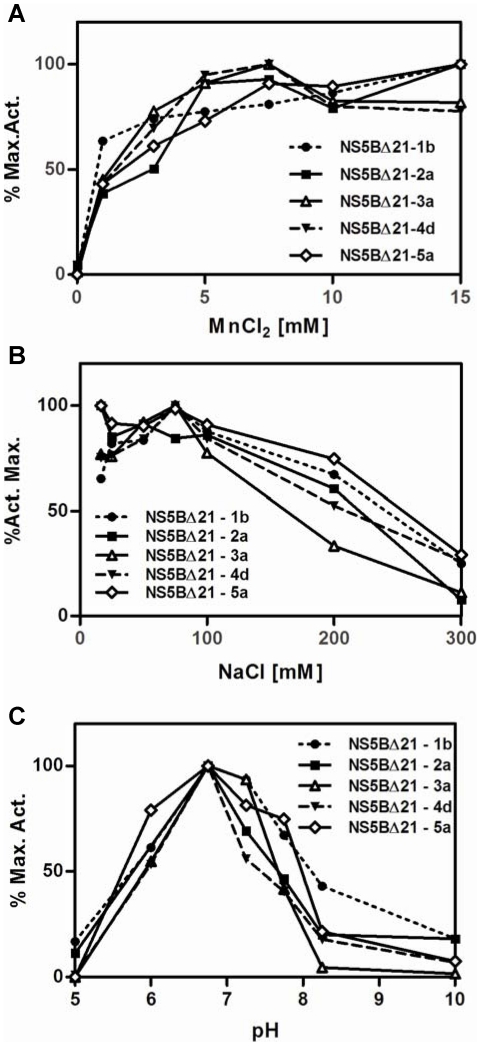
Characterization of the conditions for optimal RNA synthesis in a “de novo” initiation process by polymerases from different genotypes. The ability of each polymerase to synthesize poly-G from [α-^32^P]GTP using poly-C template is normalized with respect to its maximum activity. The effect of the following conditions was analyzed: A) MnCl_2_ concentration (metal ion requirements), B) pH and, C) NaCl concentration (ionic strength). Reaction mixtures contained 125 µM GTP and 40 ng/µl of polyC. Reactions in panel B) were performed with different buffers according to the selected pH: sodium acetate for pH 5, MES for pH 6, MOPS for pH 7.25, HEPES for pH 7.75, Tris-HCl for pH 8.25 and CAPS for pH 10. All graphs show means of at least three independent experiments. Error bars were lower than 20% in all points and have been removed for clarity.

Results for *de novo* RdRp activity at different conditions of ionic strength, manganese concentration and pH are shown in Fig. 4A, B and C, respectively. Reaction curves were normalized fixing the maximum activity for each protein to 100%. The curves obtained for each condition showed similar trends among genotypes, allowing the determination of the optimal conditions for the *de novo* RNA polymerase activity. The maximum RdRp activity was obtained at NaCl concentration below 80 mM, at high Mn^2+^ concentration (above 5 mM) and at pH around 7.

### Effect of protein concentration on polymerase activity

The NS5B polymerases from genotypes 1 to 5 were titrated to assess the effect of protein concentration on their RNA-polymerase activity. Titration data are representative of the cooperativity degree of the polymerases assayed. Data were fitted to a sigmoidal curve and the Hill coefficient was calculated from it (Figure S2). All polymerases except that of genotype 5a, showed values above 1, indicating positive cooperativity (Table 2). Values obtained from genotype 5a did not fit a sigmoidal curve and consequently we could not calculate its Hill coefficient.

### Oligomerization of NS5BΔ21

**Figure 5:**
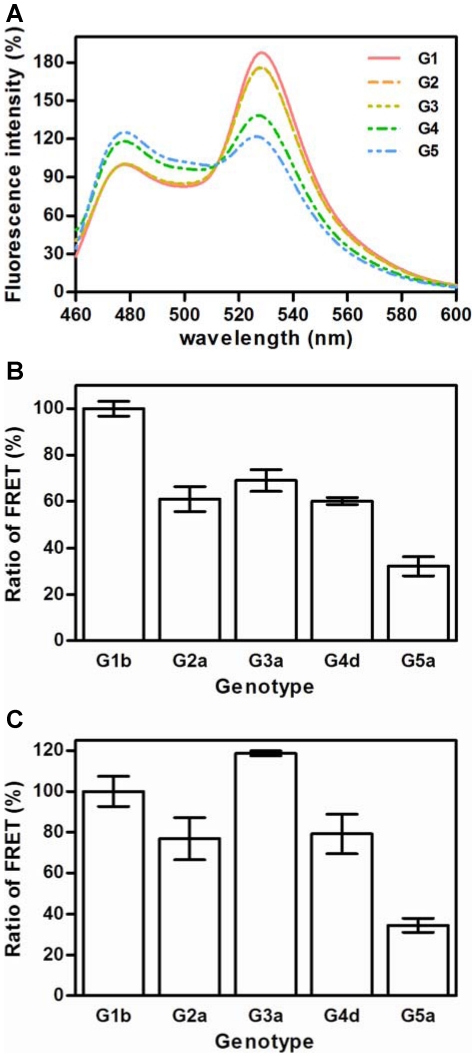
NS5B oligomerization of genotypes 1 to 5. A) Spectra obtained for a representative FRET experiment. Proteins NS5BΔ21 fused to cyan and NS5BΔ21 fused to citrine were mixed at eqimolar concentration in FRET buffer. Then, the mixture was excited at 432 nm and spectra were recorded from 460 nm to 600 nm. Two main peaks were obtained, one corresponding to cyan at approximately 478 nm and the other at approximately 530 nm corresponding to citrine. FRET ratios were calculated as the ratio of the 530 nm and 478 nm intensities. B) FRET ratios for the interaction of eqimolar amounts (50 nM each) of NS5BΔ21-cyan and NS5BΔ21-citrine for each genotype are shown. Spectra were obtained in the presence of 10 mM NaCl and 4.5 mM Mg(CH_3_COO)_2_. Values are normalized against the ratio obtained for NS5BΔ21-1b and expressed in percentage. Results are the mean and SEM of twelve independent experiments. C) FRET ratios were calculated as described above, unless spectra were obtained in the presence of 66 mM NaCl and 5 mM MnCl_2_. Ratios are normalized against the ratio obtained for NS5BΔ21-1b, and expressed in percentages. Values are the mean and SEM of six independent experiments.

Oligomerization of NS5BΔ21 from genotypes 1 to 5 was analyzed by FRET. GFP-derived fluorescent proteins cyan and citrine were fused to genotypes 1 to 5 NS5BΔ21 proteins to obtain a pair of fusion proteins per genotype. All these proteins were active (Figure S3). The genotype-specific oligomerization of the polymerases described above was analyzed by mixing equimolar concentrations of the fusion proteins (e.g., NS5BΔ21-1b-cyan and NS5BΔ21-1b-citrine) and exciting the mixtures at 432 nm. The emission spectra were recorded from 460 to 600 nm. The observation of a FRET signal (emission at 530 nm) indicated interaction between both NS5BΔ21 fusion proteins (Fig. 5A). The analysis of the obtained spectra (Fig. 5A) allowed the calculation of a simple ratio of FRET as described in Materials and Methods. The ratios of FRET obtained for each genotype at 10 mM NaCl and 4.5 mM Mg(CH_3_COO)_2_, and normalized against genotype 1 are shown in Fig. 5B. NS5BΔ21-1b showed the highest ratio of FRET, NS5BΔ21-2a, -3a and -4d showed intermediate values and the NS5BΔ21-5a showed the lowest ratio with a value below the 50% of that obtained for genotype 1. We also performed the FRET experiments under de novo activity conditions (66 mM NaCl and 5 mM MnCl_2_), and again, genotype 5 showed the lowest FRET ratio (Figure 5C). The rest of the proteins showed FRET ratio values +/− 20% of the 1b genotype (Figure 5C).

### *In silico* approach to the NS5B oligomerization study

**Figure 6:**
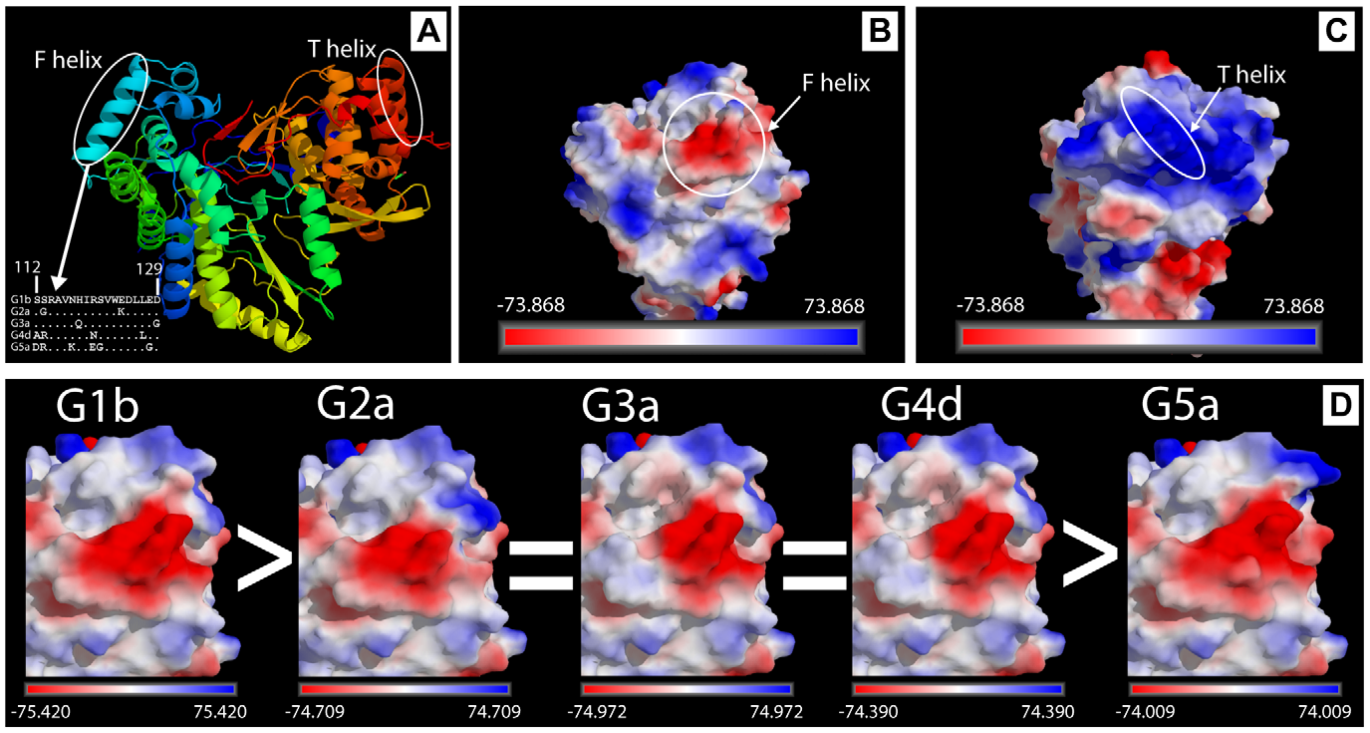
NS5B electrostatic surface potential. A) Ribbon diagram within PYMOL 1.3 [33] showing the overall structure of HCV NS5B based on the HC-J4 structure reported by Jäger and coworkers [59]. The symmetrical locations of F and T helices as well as the primary sequence corresponding to αF helix for the genotypes used in this study are shown. B) Location of electrostatic surface potential for the putative ligand site in the region corresponding to αF helix (Ser112-Asp129) of NS5B. C) Location of the electrostatic surface potential for the putative receptor site in the region corresponding to αT (Pro495-Arg505. D) Ligand electrostatic surface potential for NS5B from genotypes G1b, G2a, G3a, G4d and G5a. *In silico* mutagenesis were performed using Foldx [34] as described in Materials and Methods. The symbols > or  =  compare the intensity of electrostatic surface potential on each phenotype. For panels B, C and D, the color coded electrostatic surface potential was drawn using the Adaptative Poisson-Boltzmann equation as described in Materials and Methods [32].

Docking studies without selecting putative binding areas between receptor and ligand gave rise to different complexes that can be classified according to various criteria, such as calculating the theoretical binding energy. Based on our oligomerization data (Fig. 5), previous results [22]–[24], the abundance of crystallographic data with inhibitors bound near the αT helix (i.e. 1GX5, 1NHU, 1NHV, 1OS5, 1YVZ, 2BRL, 2D3U, 2D3Z, 2D41, 2DXS, 2GIR, 2HAI, 2HWH, 2HWI, 2I1R, 2JC0, 2O5D, 2WCX, 2WHO, 3CJ0, 3CJ2, 3CJ3, 3CJ4, 3CJ5, 3FRZ and 3HVO), and the comparison of the amino acid sequences among genotypes (Fig. 6A), allowed us to assume that regions of helixes αF (112–119) and αT (467–512) could be putative interacting sites between two NS5B where one was considered as the receptor and the other was considered as the ligand. Analysis of the electrostatic surface potential for the αF helix region (Fig. 6B) and αT helix region (Fig. 6C) showed the complementarities of surface charges. Moreover, the region corresponding to the αF helix was engineered for each genotype primary sequence by *in silico* mutagenesis using genotype 1b 2ZKU.pdb as a template, as described in Materials and Methods. The electronegativity intensity was calculated for each generated surface and the results are shown in Fig. 6D. Electronegativity was highest for genotype 1b and lowest for genotype 5a, with the following order: G1b > G2a  =  G3a  =  G4d > G5.

**Figure 7:**
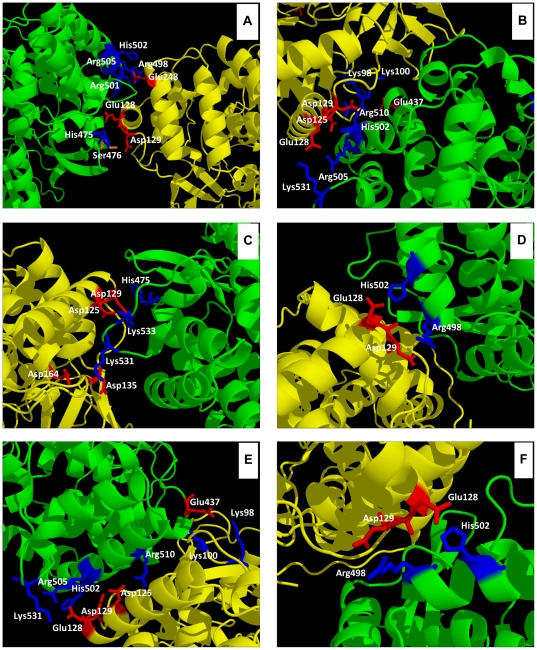
NS5B/NS5B docking model complexes showing the sites of protein-protein interaction and the amino acids involved in ionic interactions. The side chain of amino acids that form ion pairs is highlighted with red color for acidic amino acids and blue color for basic amino acids. Green and yellow colors for ribbons represent the putative ligand and receptor partners, respectively. Models 091, 004, 034, 042, 050, and 061 are represented in panels A, B, C, D, E, and F, respectively. These models were obtained as described in Materials and Methods, and Table 3.

We used GRAMM-X [35] for docking one pair of proteins (the same structure for receptor and ligand in an oligmerization process using 2ZKU.pdb) to unveil the mode of interaction across the pair [37]. Arbitrarily, we considered the region close to the αF helix as the ligand and the region near the αT helix the receptor. The docking program executes a rigid-body search using Fast Fourier Transform (FFT) correlation with simplified geometry employing shape complementarity and hydrophobicity in the scoring function [37]. Twenty-four out of 200 models were selected and refined with the FoldX software [34] to eliminate van der Waals clashes, and the theoretical binding energies were calculated. Ionic interactions and hydrogen bonding patterns in the complexes of protein-protein interactions of those refined models were determined with the Protein Interaction Calculator (PIC) [36]. We performed a second selection of 6 models with binding energies of less than or equal to -11 kcal/mol. Results are shown in Table 3 and Fig. 7.

**Table 3:** Representative parameters for docking models of NS5B.

Model name(a)	Binding energy (Kcal/mol) (b)	Protein-Protein Ionic Interactions in the NS5B(chain A)-NS5B(chain B) complex (c)	Protein-Protein Side Chain-Side Chain Hydrogen Bonds in the NS5B(chain A)-NS5B(chain B) complex: donor-acceptor (d)	Protein-Protein Main Chain-Side Chain Hydrogen Bonds in the NS5B(chain A)-NS5B(chain B) complex: donor-acceptor (d)
Model_091	−11.89	475HIS(A)-129ASP(B)	476SER(A)-125ASP(B)	476SER(A)-128GLU(B)
		498ARG(A)-248GLU(B)	476SER(A)-129ASP(B)	487SER(A)-117ASN(B)
		501ARG(A)-128GLU(B)	498ARG(A)-248GLU(B)	533LYS(A)-128GLU(B)
		502HIS(A)-248GLU(B)	502HIS(A)-248GLU(B)	248GLU(B)-498ARG(A)
		505ARG(A)-248GLU(B)	248GLU(B)-502HIS(A)	251GLN(B)-531LYS(A)
Model_004	−11.41	437GLU(A)-100LYS(B)	24ASN(A)-273ASN(B)	26LEU(A)-273ASN(B)
		437GLU(A)-98LYS(B)	505ARG(A)-128GLU(B)	514GLN(A)-100LYS(B)
		502HIS(A)-129ASP(B)	510ARG(A)-125ASP(B)	100LYS(B)-435ALA(A)
		505ARG(A)-128GLU(B)	531LYS(A)-128GLU(B)	114LYS(B)-513SER(A)
		510ARG(A)-125ASP(B)	273ASN(B)-24ASN(A)	125ASP(B)-505ARG(A)
		531LYS(A)-128GLU(B)		
Model_034	−11.01	475HIS(A)-129ASP(B)	531LYS(A)-135ASP(B)	475HIS(A)-128GLU(B)
		531LYS(A)-135ASP(B)	532THR(A)-121SER(B)	502HIS(A)-271GLY(B)
		531LYS(A)-164ASP(B)	533LYS(A)-129ASP(B)	533LYS(A)-125ASP(B)
		533LYS(A)-125ASP(B)		122VAL(B)-532THR(A)
		533LYS(A)-129ASP(B)		125ASP(B)-531LYS(A)
				259ARG(B)-531LYS(A)
				273ASN(B)-498ARG(A)
Model_042	−11.31	498ARG(A)-128GLU(B)	374HIS(A)-231ASN(B)	476SER(A)-234ARG(B)
		498ARG(A)-129ASP(B)	231ASN(B)-374HIS(A)	502HIS(A)-128GLU(B)
		502HIS(A)-128GLU(B)		65ARG(B)-380ARG(A)
				72LYS(B)-376ALA(A)
				234ARG(B)-477TYR(A)
Model_050	−11.41	437GLU(A)-100LYS(B)	24ASN(A)-273ASN(B)	26LEU(A)-273ASN(B)
		437GLU(A)-98LYS(B)	505ARG(A)-128GLU(B)	514GLN(A)-100LYS(B)
		502HIS(A)-129ASP(B)	510ARG(A)-125ASP(B)	100LYS(B)-435ALA(A)
		505ARG(A)-128GLU(B)	531LYS(A)-128GLU(B)	114LYS(B)-513SER(A)
		510ARG(A)-125ASP(B)	273ASN(B)-24ASN(A)	125ASP(B)-505ARG(A)
		531LYS(A)-128GLU(B)		
Model_061	−11.20	498ARG(A)-128GLU(B)	374HIS(A)-231ASN(B)	476SER(A)-234ARG(B)
		498ARG(A)-129ASP(B)	231ASN(B)-374HIS(A)	502HIS(A)-128GLU(B)
		502HIS(A)-128GLU(B)		65ARG(B)-380ARG(A)
				72LYS(B)-376ALA(A)
				234ARG(B)-477TYR(A)

(a) The models have been generated by the server GRAMM-X using 2zku.pdb as a template (35).

(b) Calculated with FoldX software package (34).

(c) The ionic interaction as obtained from Protein Interaction Calculator (36), showing the positions of the residue pairs in NS5B(chain A)-NS5B(chain B) docked complexes.

(d) Obtained from Protein Interaction Calculator (36).

The selected docking models share the involvement of amino acids of helices αF and αT of two different protein monomers that form variable number of ion pairs. Interactions of protein-protein side chain-side chain hydrogen bonds and main chain-side chain hydrogen bonds are responsible for the theoretically calculated binding energy. Taking this into account, the best model is the Model_091, with −11.89 kcal/mol, with up to five ion pairs between amino acids of each protein chain complex: His475-Asp129, Arg498-Glu248, Arg501-Glu128, His502-Glu248, Arg505-Glu248 (Table 3). Hydrophobic protein-protein interactions were detected in the other four models included in Table 3 except for Model_091 and Model_034. Anyhow, all models of protein-protein interaction reported here may represent allowed interactions of NS5B both *in vitro* and *in vivo*.

## Discussion

This is the first study where kinetic constants for *de novo* RdRp activity for genotypes other than 1 and 2 have been obtained. Preliminary studies of the NS5B polymerase only detected primer extension activity [10],[11]. However, more recent investigations have revealed that *in vivo* HCV RNA is replicated by a *de novo* initiation mechanism [12],[13]. This discrepancy may be due to the use of magnesium instead of manganese as the divalent metal and low concentrations of initiating nucleotide [14],[38]. We did not obtain detectable levels of *de novo* initiation using magnesium as divalent cation (data not shown). Thus, we have characterized the *de novo* NS5B activity using polyC as template and Mn^2+^ as the divalent cation. The intracellular Mn^2+^ concentration is about 100-fold lower than Mg^2+^, some authors therefore question the biological role of Mn^2+^ in NS5B replication. However, Mn^2+^ has been proposed as the preferred cation for HCV NS5B in the de novo initiation [12]. Mn^2+^ has also been proposed as the preferred cation for other flaviviridae RdRps [39]. Furthermore, Mn^2+^ has been shown to be required for Φ6 RNA polymerase activity, inducing a flexible structure that favours conformational changes [40]. On the other hand, it has been described that the apparent Kd value for the binding of Mg^2+^ to the free HCV NS5B enzyme is 3.1 mM, whereas this is 0.3 mM for Mn^2+^ ions [41]. Nevertheless, free metal ion concentrations are tightly regulated in vivo by special metal ion binding proteins, and their concentrations may vary considerably in the environment of the HCV replicative complex [42]. In the case of HCV NS5B, Mn^2+^ could be the optimal ion to stabilize the de novo conformation.

Under de novo initiation conditions, K_m_ values for GTP using NS5B from genotypes 1b and 2a (Table 2) showed comparable values (around 100 µM) to those from previous studies [16],[43]. Similar data were obtained comparing genotypes1b and 2a with genotypes 3a, 4d and 5a (K_m_ between 100 µM and 200 µM). In contrast, differences in V_max_ were higher for genotype 1b vs. the other genotypes, causing the significant variation (p-value<0.001) in efficiency among the different genotypes (Table 2). Genotype 1b showed the highest V_max_/K_m_ ratio, especially when compared to genotype 4d, which was almost 40-fold lower. In contrast, reaction conditions to obtain maximum *de novo* RNA polymerase activity were similar for all proteins (Fig. 4). Briefly, pH around 7, a final NaCl concentration around 75 mM, and MnCl_2_ final concentration above 5 mM were necessary to achieve maximum activities for all genotypes (Fig. 4). The highest activity shown by genotype 1b NS5B polymerase might be due to the presence of an Ile residue at position 405. Recently, this position has been described as a determinant for a more closed conformation, leading to a high polymerase activity and high viral kinetics [44].

The interaction that occurs among viral RdRps is critical for polymerase activity and virus proliferation [22],[45]–[48]. Recently, Kirkegaard and colleagues showed that the poliovirus RNA polymerase is required to catalyze the polymerase reaction and maintain the structure of the replication complex (RC) [49]. Indeed, the introduction of catalytically inactive polymerases into infected cells did not disrupt the formation of RCs and allowed RNA synthesis. However, the introduction of protein-protein interaction mutants inactivated RCs [49].

HCV replicates its genetic material in RCs associated with the endoplasmic reticulum membrane [50],[51], and the ratio of RNA to NS5B protein in these complexes has been calculated to be around 1 to 100 for positive strand RNA and 1 to 1000 for the replication intermediate negative strand RNA [21]. These data suggest that not all the NS5B proteins present in the HCV RC would necessarily be acting as RdRp, and some of them could play a structural role. Recent data highlight the relationship between oligomerization and *de novo* activity [52]. We have shown the oligomerization of HCV NS5B by a new FRET-based method that can detect and quantify the degree of interaction between proteins under different experimental conditions [23]. NS5B protein-protein interactions are dependent on ionic strength [23],[53]. Thus, the highest FRET values were obtained at this lowest NaCl concentration tested (10 mM) [23]. We have also demonstrated the lack of FRET signal and oligomerization of a point mutant His502Ala [24]. Thus, by evaluating HCV NS5B oligomerization in the presence of 10 mM NaCl we should expect the highest FRET values allowing for the detection of the greatest oligomerization defects. Our results indicate oligomerization differences among genotypes, even at this low NaCl concentration. The genotype 1b NS5BΔ21 protein had the highest FRET ratio (Fig. 5B), whereas, NS5BΔ21-5a showed values around 40% of the NS5BΔ21-1b. The low FRET ratio of genotype 5a NS5B showed a high correlation with the corresponding Hill coefficient result (Table 2). Furthermore, we have performed FRET experiments under de novo reaction conditions (66 mM NaCl and 5 mM MnCl_2_). Under these conditions, the obtained results show a relationship with the Hill coefficient values (compare Table 2 and Figure 5C). Also under these conditions, genotype 5 showed the lowest FRET ratio.

It was intriguing to know if there is a relationship between the polymerase sequence and cooperativity and oligomerization capacity. Although the heterogeneity of the sequences analyzed in this study is high (Fig. 1 and Table 1), only structural differences at NS5B regions exposed to the solvent [54]–[59] and previously related to NS5B-NS5B interactions were considered (Table 2 and Fig. 5). According to the crystal structure 2ZKU.pdb, α-helixes C (residues 62 to 78), F (residues 112 to 129), K (residues 231 to 242), L (residues 247 to 260) and N (residues 329 to 346) are possible sites of interaction with αT helix (His502). However, none of them showed correlation between sequence changes and oligomerization, except for the αF helix. The total number of changes in the αF helix increased as oligomerization decreased (Fig. 6A). Furthermore, the electronegative surface potential intensity clearly decreased in parallel to a decrease in protein oligomerization (Fig. 6D). Intriguingly, changes found in genotype 5 NS5B are in the same position relative to the helix turn and facing the most external part of the helix, leading to changes in the overall electronegativity of the helix (Fig. 6B and 6D). The abundance of Asp and Glu residues confers an electronegative surface potential to the αF helix and makes this region a putative partner of protein-protein interaction dominated by electrostatic interactions. Simulation data showed that position Glu248 was important for protein-protein interactions (Table 3 and Fig. 7), forming together with the αF helix residues Glu128 and Asp129 a discontinuous epitope involved in NS5B-NS5B interactions. The second partner for these interactions may be the region of αT helix where His502 is located. This region has a high electropositive surface potential intensity (see Fig. 6C) due to the number of basic residues present. Some residues defined in Table 3 and Fig. 7 had been previously related to oligomerization [22],[24],[55]. Fig. 1 shows that while the αF helix has many mutations that alter their negative charge density, the αT helix is very similar for all genotypes and mutations are rare and very conservative. However, genotype 2 contains the mutation His502Ser, and the cooperativity and oligomerization values were high and almost similar to that of genotype 1. This His502Ser mutation could be compensated with changes Pro247Glu, Arg250His, and Arg254His, all of them found in the immediacy of Glu248. On the other hand, genotype 5 protein contains mutation Glu128Gly, but in this case there are no compensatory mutations in helix T. This might be the reason why genotype 5 NS5B showed the lowest oligomerization values.

A number of crystal structures of the HCV polymerase (i.e. 1GX5, 1NHU, 1NHV, 1OS5, 1YVZ, 2BRL, 2D3U, 2D3Z, 2D41, 2DXS, 2GIR, 2HAI, 2HWH, 2HWI, 2I1R, 2JC0, 2O5D, 2WCX, 2WHO, 3CJ0, 3CJ2, 3CJ3, 3CJ4, 3CJ5, 3FRZ and 3HVO), show that the αT helix is close to a site of interaction for non-nucleoside inhibitors. When comparing the secondary structure of various crystals of NS5B/NNI complexes the overall structure clearly remains relatively unchanged upon inhibitor binding, except for positions in the region Pro495-Arg505 [25]. Biswal and coworkers [25] explained the mechanism of action of thiophene-based inhibitors in two directions: first, the structural shifts of αT helix affect the integrity of the GTP binding site, resulting in reduced affinity for GTP and contributing to the formation of an RNA polymerase state incapable of carrying out a polymerization cycle. Secondly, the NS5B may need to oligomerize in order to function. His502 is one of the critical residues involved in homomeric interaction levels and mutants His502Glu and His502Ala abolish the enzymatic activity [24]. The position of His502 in the NNI bound-state is greatly perturbed suggesting that non-nucleoside inhibitors could have profound effect on the oligomerization process [25]. Additionally, it seems clear that variations in the negative charge density of the αF helix among different genotypes should also help to explain the variations in the degree of oligomerization. Ongoing work should define the role played by these two NS5B regions in the oligomerization process. Results in this study have not allowed us to establish a clear relationship between activity and oligomerization. Site-directed mutagenesis of these two regions will be very useful to understand the role of oligomerization, if any, in de novo initiation, primer extension and template switching activities. Such experiments will also help to establish a better understanding of the differences observed in FRET ratios when comparing different reaction conditions (Figures 5B and 5C).

The efficacy of current HCV treatment regimens and those in current clinical trials is dependent on the HCV genotype. Understanding functional and structural differences among HCV genotypes is crucial for the design of new therapies. Hampering the interaction among NS5B polymerases has been proposed as a mechanism of action for drugs under development [23]–[26]. Our study provides, for the first time, evidence of the *de novo* RNA-polymerase activity and oligomerization of NS5B from genotypes different to genotypes 1 and 2 and propose candidate regions involved in NS5B-NS5B interactions. A better understanding of the replication process and how this is affected by genotypic differences may allow the development of effective treatments against all the HCV genotypes.

## Supporting Information

Figure S1 **Electro-mobility shift assay.** A) Native polyacrylamide gel showing the RNA free probe (lane 1) as well as retarding products for genotype 1 (lanes 2 and 3), genotype 2 (lanes 4 and 5), genotype 3 (lanes 6 and 7), genotype 4 (lanes 8 and 9), and genotype 5 (lanes 10 and 11). The NS5B concentration was fixed to 0.6 µM (lanes 2, 4, 6, 8, and 10) and 1.8 µM (lanes 3, 5, 7, 9, and 11). B) Representation of the ratio of protein-bound to RNA free probe (in percentage). For each genotype and each protein concentration used (0.6 and 1.8 µM) the percentage of the free RNA probe (in white) and the protein-bound RNA probe (in black) are represented. C) Comparison between V_max_/K_m_ data from Table 2 and those data normalized using the ratios obtained from the electro-mobility shift assay.
(PPT)

Figure S2 **Hill coefficient data.** Panels A, B, C, D, and E represent the kinetic data for NS5B from genotypes 1, 2, 3, 4, and 5, respectively. The r2 values are shown for all graphics. The best fit of the experimental data was to a sigmoidal curve, as described in Materials and Methods, except for genotype 5, which did not fit to a sigmoidal curve and instead produced a better fit to a linear curve.
(PPT)

Figure S3 **Activity of NS5B-fused proteins.** LE19 oligonucleotide was used as the template and de novo polymerase activity was analyzed in polymerase buffer (MOPS 20 mM, NaCl 66 mM, MnCl_2_ 5 mM) in the presence of 125 µM NTPs, and 0.5 µCi of α[^32^P]GTP (3000 Ci/mmol, PerkinElmer). Reactions were initiated by the addition of 600 nM purified NS5B and incubated at 25°C. After one hour of incubation reactions were stopped and products were resolved in a polyacrylamide gel and visualized by phosphorimaging.
(PPT)

Table S1 **Primers used in the study.**
(DOC)
