# Supplementary material for: De Novo Polymerase Activity and Oligomerization of Hepatitis C Virus RNA-Dependent RNA-Polymerases from Genotypes 1 to 5
Source: PLoS One. 2011 Apr 7;6(4):e18515. doi: 10.1371/journal.pone.0018515 (PMC3072391; doi:10.1371/journal.pone.0018515)
Supplement: Figure S1 — Electro-mobility shift assay. A) Native polyacrylamide gel showing the RNA free probe (lane 1) as well as retarding products for genotype 1 (lanes 2 and 3), genotype 2 (lanes 4 and 5), genotype 3 (lanes 6 and 7), genotype 4 (lanes 8 and 9), and genotype 5 (lanes 10 and 11). The NS5B concentration was fixed to 0.6 µM (lanes 2, 4, 6, 8, and 10) and 1.8 µM (lanes 3, 5, 7, 9, and 11). B) Representation of the ratio of protein-bound to RNA free probe (in percentage). For each genotype and each protein concentration used (0.6 and 1.8 µM) the percentage of the free RNA probe (in white) and the protein-bound RNA probe (in black) are represented. C) Comparison between Vmax/Km data from Table 2 and those data normalized using the ratios obtained from the electro-mobility shift assay. (PPT) [file pone.0018515.s001.ppt]

## Slide 1
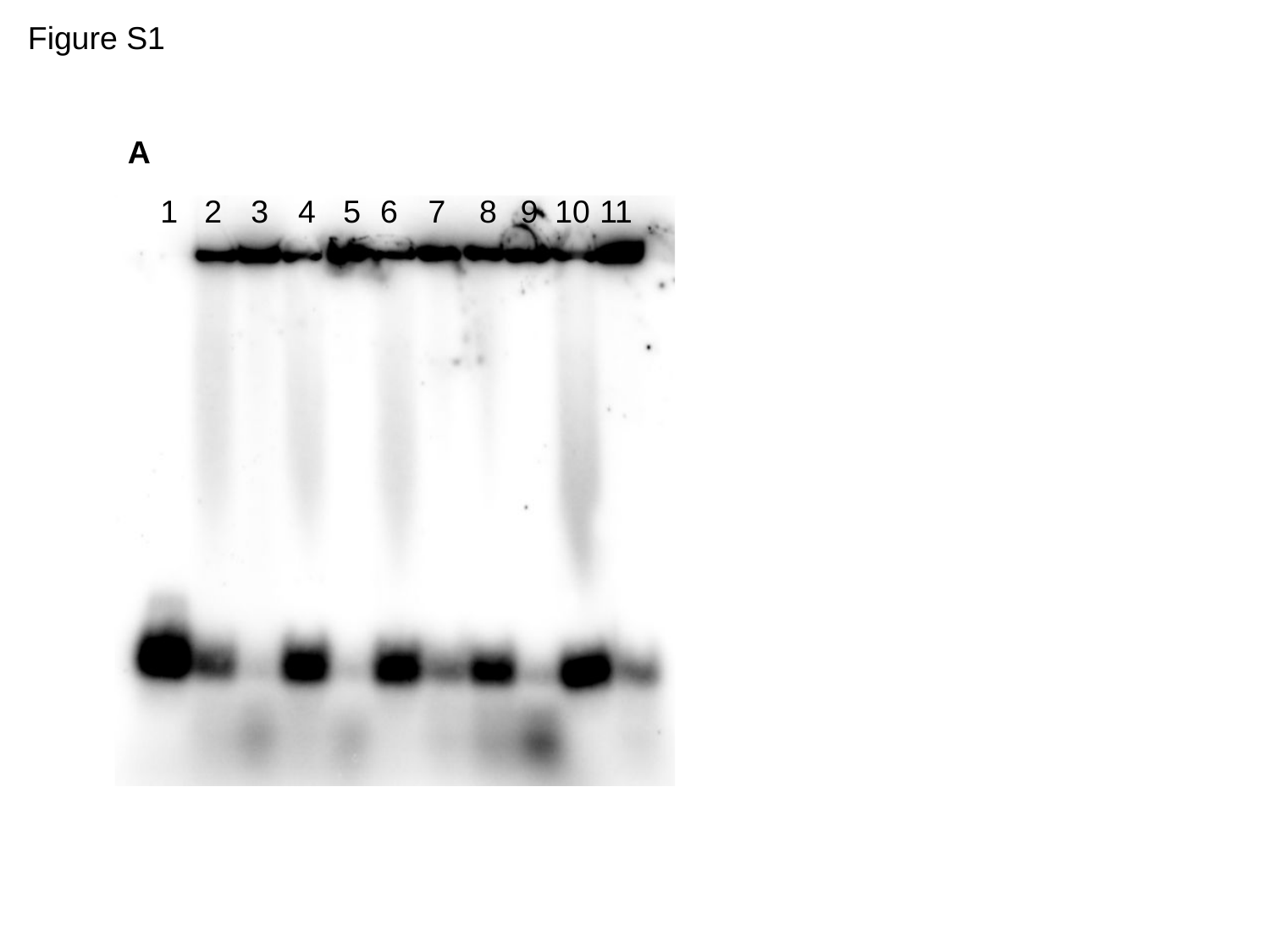

Figure S1
A
1
2
3
4
5
6
7
8
9
10
11

## Slide 2
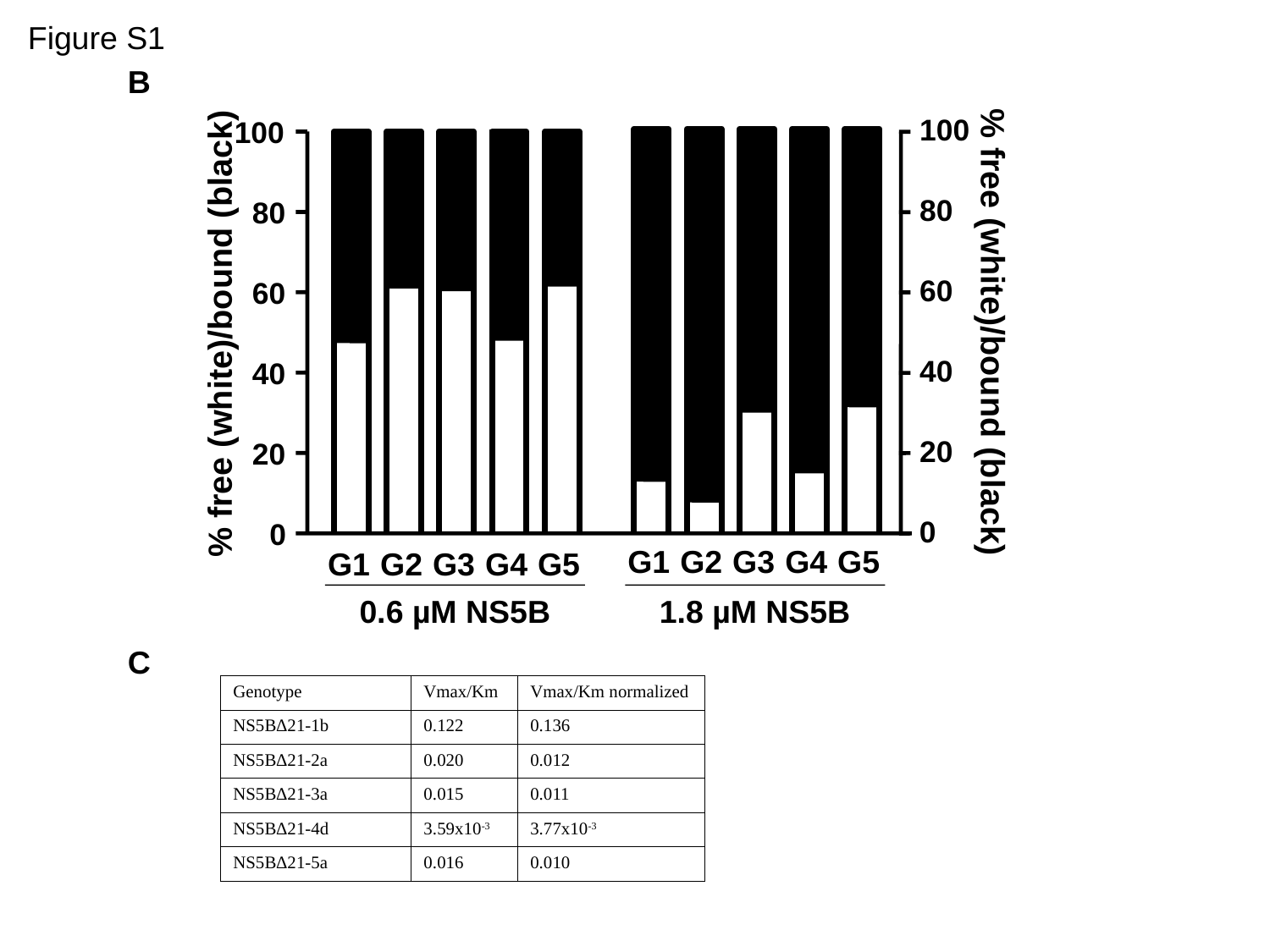

Figure S1
B
100
100
80
80
60
60
% free (white)/bound (black)
% free (white)/bound (black)
40
40
20
20
0
0
G1
G2
G3
G4
G5
G1
G2
G3
G4
G5
0.6 µM NS5B
1.8 µM NS5B
C
| Genotype | Vmax/Km | Vmax/Km normalized |
| --- | --- | --- |
| NS5BΔ21-1b | 0.122 | 0.136 |
| NS5BΔ21-2a | 0.020 | 0.012 |
| NS5BΔ21-3a | 0.015 | 0.011 |
| NS5BΔ21-4d | 3.59x10^-3^ | 3.77x10^-3^ |
| NS5BΔ21-5a | 0.016 | 0.010 |
