# Supplementary material for: De Novo Polymerase Activity and Oligomerization of Hepatitis C Virus RNA-Dependent RNA-Polymerases from Genotypes 1 to 5
Source: PLoS One. 2011 Apr 7;6(4):e18515. doi: 10.1371/journal.pone.0018515 (PMC3072391; doi:10.1371/journal.pone.0018515)
Supplement: Figure S2 — Hill coefficient data. Panels A, B, C, D, and E represent the kinetic data for NS5B from genotypes 1, 2, 3, 4, and 5, respectively. The r2 values are shown for all graphics. The best fit of the experimental data was to a sigmoidal curve, as described in Materials and Methods, except for genotype 5, which did not fit to a sigmoidal curve and instead produced a better fit to a linear curve. (PPT) [file pone.0018515.s002.ppt]

## Slide 1
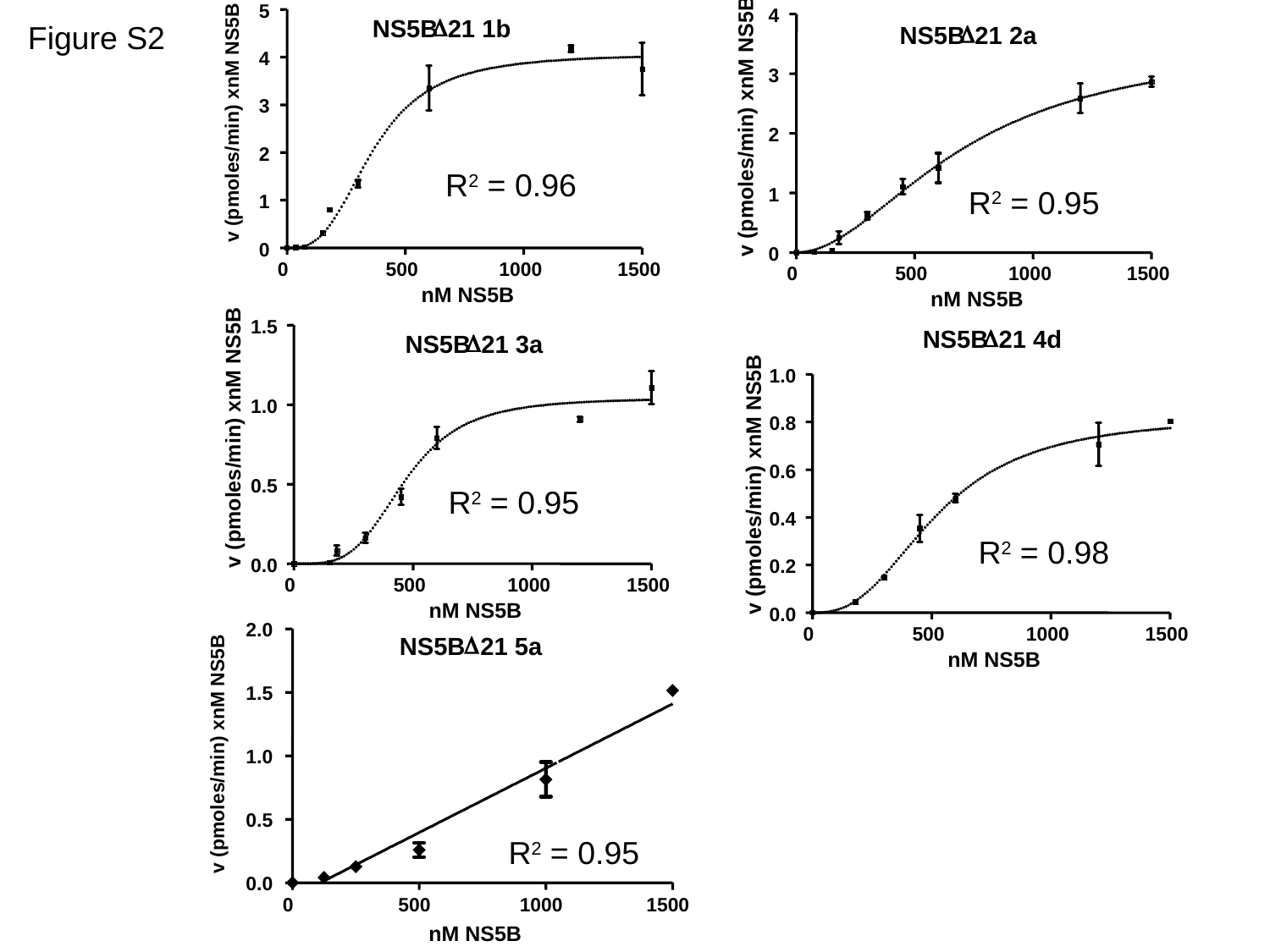

4
Δ
NS5B
21 2a
3
v (pmoles/min) xnM NS5B
2
1
0
0
500
1000
1500
nM NS5B
R2 = 0.95
5
Δ
21 1b
NS5B
4
3
v (pmoles/min) xnM NS5B
2
1
0
0
500
1000
1500
nM NS5B
R2 = 0.96
Figure S2
Δ
NS5B
21 4d
1.0
0.8
0.6
v (pmoles/min) xnM NS5B
0.4
0.2
0.0
0
500
1000
1500
nM NS5B
R2 = 0.98
1.5
Δ
21 3a
NS5B
1.0
v (pmoles/min) xnM NS5B
0.5
0.0
0
500
1000
1500
nM NS5B
R2 = 0.95
2.0
Δ
NS5B
21 5a
1.5
v (pmoles/min) xnM NS5B
1.0
0.5
0.0
0
500
1000
1500
R2 = 0.95
nM NS5B
