# Supplementary figures and images for: De Novo Polymerase Activity and Oligomerization of Hepatitis C Virus RNA-Dependent RNA-Polymerases from Genotypes 1 to 5
Source: PLoS One. 2011 Apr 7;6(4):e18515. doi: 10.1371/journal.pone.0018515 (PMC3072391; doi:10.1371/journal.pone.0018515)

## Slide 1
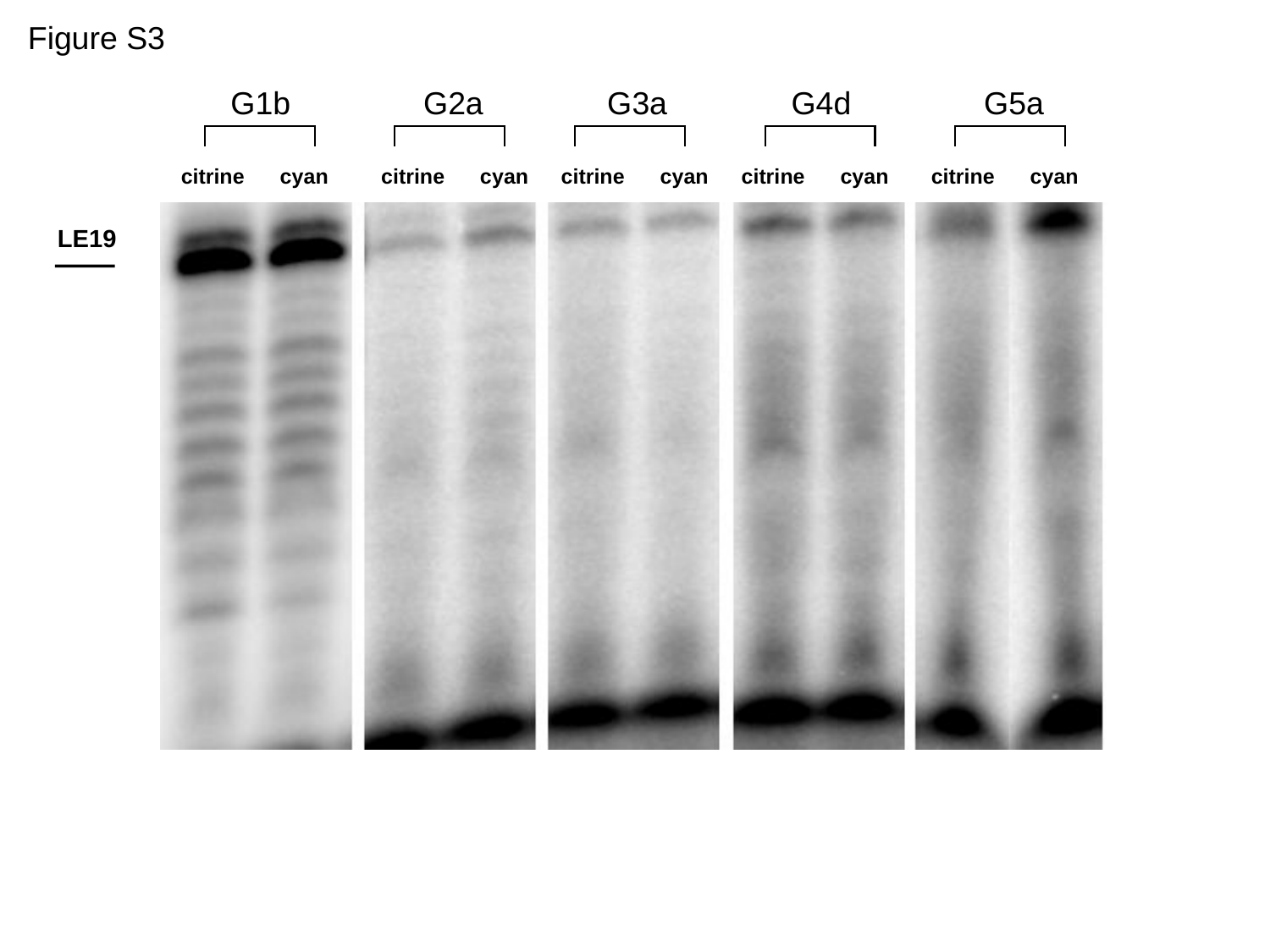

Figure S3
 G1b G2a G3a G4d G5a
 citrine cyan
 citrine cyan
 citrine cyan
 citrine cyan
 citrine cyan
LE19

Supplement: Figure S3 — Activity of NS5B-fused proteins. LE19 oligonucleotide was used as the template and de novo polymerase activity was analyzed in polymerase buffer (MOPS 20 mM, NaCl 66 mM, MnCl2 5 mM) in the presence of 125 µM NTPs, and 0.5 µCi of α[32P]GTP (3000 Ci/mmol, PerkinElmer). Reactions were initiated by the addition of 600 nM purified NS5B and incubated at 25°C. After one hour of incubation reactions were stopped and products were resolved in a polyacrylamide gel and visualized by phosphorimaging. (PPT) [file pone.0018515.s003.ppt]
