# Supplementary material for: De Novo Polymerase Activity and Oligomerization of Hepatitis C Virus RNA-Dependent RNA-Polymerases from Genotypes 1 to 5
Source: PLoS One. 2011 Apr 7;6(4):e18515. doi: 10.1371/journal.pone.0018515 (PMC3072391; doi:10.1371/journal.pone.0018515)
Supplement: Table S1 — Primers used in the study. (DOC) [file pone.0018515.s004.doc]

Table S1.

| **Primer** | **Process** | **Sequence (5’→3’)** |
| --- | --- | --- |
| HCV3a-RT | RT/ePCR/nPCR | GAAATGGAGTGTTATCCTACC |
| HCV4a-RT | RT/ePCR/nPCR | CCTAAGGTCGGAGTGTTAAG |
| HCV5a-RT | RT/ePCR/nPCR | GGAGTGTTTAGCTCCCAGC |
| WF33 | ePCR | ACGCAGAAAGCGTCTAGCCAT |
| NS5BFL-3F | nPCR | CTCTTGGTCCACCGTTAGTG |
| NS5BFL-4F | nPCR | ACATCAGATTCTTGGTCCAC |
| NS5BFL-5F | nPCR | CGGACGCGGCTTCATATTCTTCC |
| G1Attb1 | ∆21PCR/ifPCR/fPCR | GGGGACAAGTTTGTACAAAAAAGCAGGCTTCTAAGGAGGTAGAACCATGAAATCAATGTCCTACACATGGACAGG |
| G1∆21Attb2 | ∆21PCR | GGGGACCACTTTGTACAAGAAAGCTGGGTCCTAATGGTGATGGTGATGGTGGCGGGGTCGGGCACGAGACAGGC |
| G2Attb1 | ∆21PCR/fPCR/fPCR | GGGGACAAGTTTGTACAAAAAAGCAGGCTTCTAAGGAGGTAGAACCATGAAATCCATGTCATACTCCTGGACC |
| G2∆21Attb2 | ∆21PCR | GGGGACCACTTTGTACAAGAAAGCTGGGTCCTAATGGTGATGGTGATGGTGGCGGGGTCGGGCACGCGACACGC |
| G3Attb1 | ∆21PCR/ifPCR/fPCR | GGGGACAAGTTTGTACAAAAAAGCAGGCTTCTAAGGAGGTAGAACCATGAAATCTATGTCGTACTCTTGGAC |
| G3∆21Attb2 | ∆21PCR | GGGGACCACTTTGTACAAGAAAGCTGGGTCCTAATGGTGATGGTGATGGTGGCGGGTTCGGGCACGTGACACGC |
| G4Attb1 | ∆21PCR | GGGGACAAGTTTGTACAAAAAAGCAGGCTTCTAAGGAGGTAGAACCATGAAATCAATGTCCTACTCGTGGACG |
| G4∆21Attb2 | ∆21PCR/ifPCR/fPCR | GGGGACCACTTTGTACAAGAAAGCTGGGTCCTAATGGTGATGGTGATGGTGGCGGGGTCGGGCATGGGACACGC |
| G5Attb1 | ∆21PCR | GGGGACAAGTTTGTACAAAAAAGCAGGCTTCTAAGGAGGTAGAACCATGAAATCCATGTCATACAGCTGGAC |
| G5∆21Attb2 | ∆21PCR/ifPCR/fPCR | GGGGACCACTTTGTACAAGAAAGCTGGGTCCTAATGGTGATGGTGATGGTGGCGGGGTCGGGCACGGGACATGC |
| G1∆21-TEV | ifPCR | ACCCTGGAAGTACAGGTTTTCGCGGGGTCGGGCACGAGACAGGC |
| G2∆21-TEV | ifPCR | ACCCTGGAAGTACAGGTTTTCGCGGGGTCGGGCACGCGACACGC |
| G3∆21-TEV | ifPCR | ACCCTGGAAGTACAGGTTTTCGCGGGTTCGGGCACGTGACACGC |
| G4∆21-TEV | ifPCR | ACCCTGGAAGTACAGGTTTTCGCGGGGTCGGGCATGGGACACGC |
| G5∆21-TEV | ifPCR | ACCCTGGAAGTACAGGTTTTCGCGGGGTCGGGCACGGGACATGC |
| TEV-GFP | ifPCR | GAAAACCTGTACTTCCAGGGTGTGAGCAAGGGCGAGGAGCTGTTCA |
| GFP-Attb2 | ifPCR/ fPCR | GGGGACCACTTTGTACAAGAAAGTCGGGTCCTAATGGTGATGGTGATGGTGCTTGTACAGCTCGTCCATGCCG |

RT, retrotranscription (cDNA)

ePCR, external PCR (whole coding region)

nPCR, nested PCR (whole NS5B)

∆21PCR, PCR of NS5B∆21 (Gateway cloning)

ifPCR, individual fusion PCR (NS5B∆21 or cyan/citrine)

fPCR, fusion PCR (NS5B∆21+cyan/citrine for Gateway cloning)
